# Avoidable mortality for people with disabilities: a systematic review and meta-analysis

**DOI:** 10.1136/bmjph-2025-004826

**Published:** 2026-10-05

**Authors:** Sara Rotenberg, Yi Yang, Maureen Moyo-Chilufya, Jane Wilbur, Tracey Smythe, Hannah Kuper

**Affiliations:** 1 International Centre for Evidence in Disability, Department of Population Health, London School of Hygiene & Tropical Medicine, London, UK; 2 The University of Melbourne School of Population and Global Health, Melbourne, Victoria, Australia; 3 Stellenbosch University, Stellenbosch, South Africa

**Keywords:** Epidemiology, Public Health, Systematic Review

## Abstract

**Objectives:**

To estimate the proportion and relative inequalities in avoidable mortality for people with disabilities globally.

**Design:**

Systematic review and meta-analysis of population-based studies.

**Data sources:**

A systematic search of Embase, MEDLINE, Global Health and PsycINFO was conducted for articles on disability and mortality published from January 2000 to July 2026.

**Eligibility criteria:**

We included studies that reported on avoidable, treatable and preventable mortality and disability. Eligible studies were quantitative studies that provided a numeric proportion of avoidable mortality and/or relative measures compared with people without disabilities.

**Results:**

Our search identified 1250 deduplicated records that were assessed for eligibility and 12 articles representing 15 unique cohorts were included in the study. All the data were from high-income countries and examined avoidable mortality for people with intellectual and developmental disabilities. Most were low risk of bias (75%) and the rest were medium (25%), as measured by the JBI Quality Appraisal Tool for Cohort Studies. Avoidable mortality was reported overall and for treatable and preventable sub-indicators. Results had high heterogeneity, but found people with disabilities had 45% (95% CI 45% to 46%) of deaths deemed avoidable, 22% (95% CI 21% to 22%) treatable and 21% (95% CI 20% to 21%) preventable. This represented significant relative inequalities for 3.60 (95% CI 3.53 to 3.68) times higher avoidable mortality; 5.82 (95% CI 5.64 to 6.01) times higher treatable mortality and 2.58 (95% CI 2.50 to 2.66) times higher preventable mortality compared with people without disabilities.

**Conclusions:**

A substantial proportion of early mortality for people with disabilities is likely to be avoidable. Some of this may be due to underlying impairments, but these inequalities are unlikely due to impairment alone. Rather, they reflect significant health inequalities that contribute to differences in life expectancy compared with people without disabilities, though all data are from high-income countries. Health systems must adapt to improve accessibility and quality of care for this population.

**PROSPERO registration number:**

CRD420251072430.

**WHAT IS ALREADY KNOWN ON THIS TOPIC**

- People with disabilities have higher mortality than people without disabilities.
- Studies from Europe have shown elevated levels of avoidable mortality among people with intellectual disabilities, but no global synthesis of the evidence on avoidable mortality across people with disabilities has previously been available.

**WHAT THIS STUDY ADDS**

- Our meta-analysis shows that people with intellectual and developmental disabilities are more than three times as likely to experience avoidable mortality as people without disabilities, and that approximately 45% of these deaths are avoidable.
- We also found a lack of evidence from other disability groups and from low- and middle-income countries, limiting understanding of the global burden of avoidable mortality for people with disabilities.

**HOW THIS STUDY MIGHT AFFECT RESEARCH, PRACTICE OR POLICY**

- Improving timely access to high-quality healthcare and strengthening health system accountability may help reduce avoidable deaths for people with disabilities, while further research is needed to generate evidence for other disability groups and settings, particularly in low- and middle-income countries.

## Background

 There are 1.3 billion people with disabilities globally. People with disabilities constitute a heterogenous group of people, but are united in experiencing barriers to full and equal participation because of their impairments and environment.^1^ Consequently, people with disabilities are known to experience a variety of inequities in educational attainment, employment, poverty and social development—all of which are important social determinants of health.^2^ In addition, evidence demonstrates that the health sector is a particular area where people with disabilities experience inequities, largely due to barriers to accessing healthcare and unmet needs for services.^2^ The well-understood barriers, like poor health worker knowledge around disability, inaccessible facilities and communication, and poor financial protection, contribute to worse access and outcomes for people with disabilities.^3^ In addition, despite having greater healthcare needs related to underlying impairments, people with disabilities consistently experience significantly higher levels of unmet need than people without disabilities.^4–6^

Adverse social determinants of health, barriers to health access and poorer health status ultimately culminate in worse life expectancy for people with disabilities. A global systematic review and meta-analysis showed that people with disabilities have a 2.2-fold higher mortality rate, equating to a 13.8-year difference in life expectancy.^7^ These findings hold across country income levels, with similar differences in mortality rates in low- and middle-income countries,^8,9^ though there are pronounced differences by age and impairment type. For example, a recent systematic review showed children with disabilities aged 0–19 have a 5.31 times higher mortality rate,^10^ compared with 1.96 times higher in older adults.^7^ Evidence shows that this also differs by impairment type, with higher life expectancy gaps for people with psychosocial disabilities and intellectual disabilities.^11,12^

A portion of the early mortality experienced by people with disabilities may be related to underlying impairments or health conditions. For example, people with Down syndrome are predisposed to certain types of blood cancer, congenital heart disease and respiratory infections,^13^ and people with spinal cord injuries may have underlying respiratory problems that make them more susceptible to mortality from respiratory infections.^14^ However, there is emerging evidence from the UK, Denmark, Sweden and Canada that people with disabilities may experience a high proportion of avoidable mortality.^12,15–18^ For example, in the UK, the Learning Disability Mortality Review (LeDeR) programme has shown people with learning disabilities die nearly 20 years earlier and that approximately 40% of these deaths are avoidable—a frequency twice that of the general population.^19,20^ Additionally, there is increasing evidence that people with disabilities may have higher rates of cause-specific mortality that could be prevented, identified or treated with better and more equitable public health interventions and access to healthcare.^7^ Avoidable mortality is a particularly relevant indicator because it captures deaths that are considered through public health interventions or early diagnosis and treatment.^21^ This includes certain cancers (eg, prostate, cervical, breast, gastric, lung),^22–28^ cardiovascular disease,^29^ alcohol-related deaths^30^ and respiratory infections like COVID-19.^31^ However, there remain gaps in synthesised evidence on avoidable mortality for people with disabilities. Consequently, the aim of this study was to conduct a systematic review and meta-analysis on the prevalence and relative inequities of avoidable mortality for people with disabilities compared with people without disabilities.

## Methods

### Definitions

Disability was defined using the United Nations Convention on the Rights of Persons with Disabilities, which defines people with disabilities as ‘those who have long-term physical, mental, intellectual or sensory impairments which in interaction with various barriers may hinder their full and effective participation in society on an equal basis with others’. This definition includes people with specific conditions deemed likely to result in disability (eg, spina bifida, muscular dystrophy, Down syndrome, etc), specific impairments (eg, visual, hearing, physical) as well as disability measured through functioning/activity limitations/self-report (eg, Washington Group questions, activities of daily living). We excluded those with mild functional impairments, frailty or specific diseases that have a range of functioning and therefore are not necessarily categorised as disability (eg, Parkinson’s, anxiety, depression, etc).

According to Organisation for Economic Co-operation and Development (OECD), avoidable deaths are those that are considered either to be preventable (by better accessibility, availability and quality of care and public health and preventative actions) or treatable (with timely and effective care; also known as amenable mortality).^32,33^ Avoidable mortality is reported overall and by treatable and preventable sub-indicators. It is usually only measured in people under 75 years, though some countries include follow-up until 80 years.

### Inclusion and exclusion criteria

We included studies that reported on avoidable mortality and disability. Eligible studies were quantitative studies that provided a numeric proportion of avoidable mortality among people with disabilities and relative inequalities compared with a control group of people without disabilities. We included studies conducted in large datasets, coroner reports or other valid mortality data sources (eg, verbal autopsy). Studies were excluded if they did not have a valid measure of disability or avoidable mortality, in line with the inclusion criteria. A full list of reasons for exclusion is found in online supplemental appendix 5.

### Search strategy

A systematic search of Embase, MEDLINE, Global Health and PsycINFO was conducted for articles on disability and mortality published from 1 January 2000 to the date of search (21 July 2026) by SR. Search terms were piloted in the Ovid search platform and can be found in online supplemental appendix 1. The search process and study protocol were registered on PROSPERO in July 2025 (CRD420251072430) and followed the Preferred Reporting Items for Systematic Reviews and Meta-Analyses (PRISMA) checklist (online supplemental appendix 4). Grey literature may have been included in the databases, but was not explicitly searched, while government reports were not systematically searched due to the global nature of the review. Reference tracing was done, but did not yield any additional studies.

### Screening and extraction

Independent title, abstract and full-text screening was conducted by SR and YY in Covidence with high levels of agreement (screening Cohen’s kappa 0.844; full-text Cohen’s kappa: 0.797). All screening conflicts were included in the full-text review, due to the small number of conflicts and full-text conflicts were resolved by discussion. A data extraction sheet was developed in Microsoft Excel and piloted on three studies. Information on the study design, demographics and exposure and outcome measures were extracted (online supplemental appendix 2). SR extracted all relevant data from the papers, which was checked by YY. While most studies reported on sex in demographics tables, there were no sex-disaggregated analyses and so this was not extracted.

### Risk of bias

Risk of bias for included studies was assessed using the Joanna Briggs Institute (JBI) Critical Appraisal Checklist for Cohort Studies by YY and checked by SR (online supplemental appendix 3). While the protocol initially reported using the Newcastle-Ottawa Scale, we used the JBI checklist as that was considered more appropriate for assessing the quality of included population-based cohort studies. The tool has 11 questions to assess potential sources of bias in study design. Studies were rated as low risk of bias, moderate and high risk of bias.

### Meta-analysis

All statistical analyses were conducted in R (V.4.4.1) for each of the main outcomes (avoidable mortality) and sub-indicators (preventable and treatable mortality) and exposure status (disabled or non-disabled). Meta-analyses were conducted on both reported proportions and relative measures. First, we used Wang’s^34^ approach for conducting meta-analyses of proportions with double arcsine transformation and inverse sample size weighting to account for differences in sample sizes to create an overall proportion across studies. We manually calculated any data on missing sample sizes or proportions based on the information provided (eg, for Hirvikoski *et al*^16^ we divided the number of avoidable deaths by the total number of deaths using a formula in Excel to get a proportion). Next, relative measures (HRs, ORs, relative risks (RRs)) were pooled using common effects meta-analysis methods, where they were reported. We treated HR, standardised mortality ratios, RR and ORs as relative effect measures comparable to HRs under the rare-outcome assumption.^35^ Most studies reported unadjusted relative measures, and so these were used for the meta-analysis. Included studies were highly comparable in terms of study design, population, disability definitions, outcomes and analytical approaches; therefore, a common-effect model was used to estimate a shared underlying effect.^36^ Sensitivity analyses using random-effects models were calculated and produced similar estimates of proportions for people with disabilities and relative inequalities outcomes. For people without disabilities, pooled estimates differed owing to the greater influence of smaller comparison groups with higher prevalence estimates (online supplemental appendix 6).

Studies that only reported on proportions or relative measures were excluded from the respective meta-analyses. Different cohorts within the same study were included as separate populations for the meta-analyses,^16^ except for Heslop *et al*, where the population-based cohort was selected instead for greater representativeness.^12^ The multiple included UK studies were checked for overlap, and most had different time frames, included countries or age groups precluded overlap. However, Glover *et al* and Hosking *et al* were deemed to have potential overlap for their common outcome (proportion of avoidable mortality), so Glover *et al* was selected for its recency. Hosking *et al* was still included, as it also reported proportions of preventable and treatable mortality as well as relative inequalities for all three outcomes that were not reported by Glover *et al*.

### Role of funders and patient and public involvement

The funders had no role in the study design, analysis or write-up of results. No patients or public members were involved in the design of the study, though some of the authors have personal or family experience of disability.

## Results

Our search identified 3072 records, of which 1250 were included for screening after deduplication (n=1822). Following the title and abstract screening, 1150 records were excluded and a further 84 studies were excluded after full-text review (figure 1). We excluded a further two studies at the extraction phase, and could not retrieve two full texts. Ultimately, there were 12 eligible studies (online supplemental appendix 3) that included 15 unique cohorts. While Heslop *et al* reported a population-based and random sub-group estimate, only the population-based estimate was included in this study.

**Figure 1:**
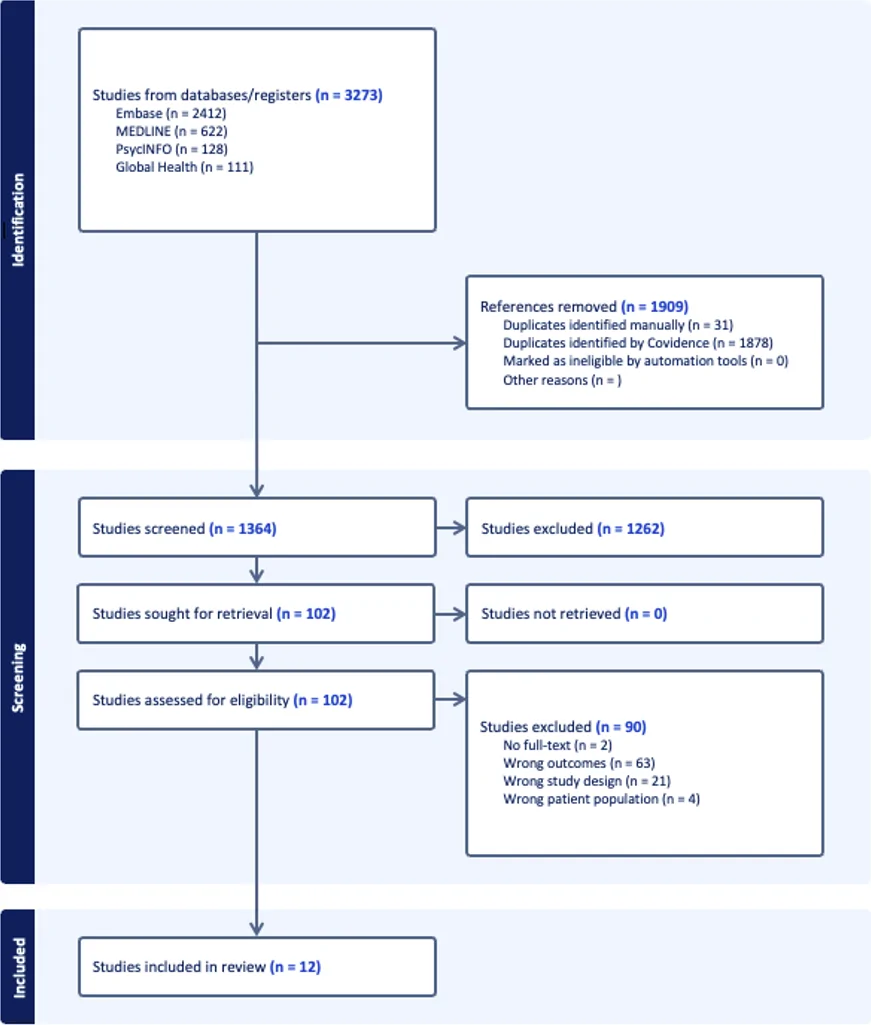
Preferred Reporting Items for Systematic Reviews and Meta-Analyses (PRISMA) diagram.

All studies were conducted in high-income countries in Europe and North America, including the UK (58%; n=7), Canada (17%; n=2), Sweden, Denmark and Australia (all 8%; n=1) (table 1). All included studies were focused on intellectual disability only (83%; n=10) or intellectual and developmental disability (17%; n=2). Most studies used a disability register as the method for assessing disability status (67%; n=8) and all used population-based sources of data, such as general practitioner (GP) records or administrative population datasets. Avoidable mortality was defined by OECD or national statistics offices definitions that were either comparable with or used OECD definitions; only one study included its own code list based on previous studies that used the OECD framework. The mean follow-up time was 8.3 years and the median was 5 years, while the mean sample size was 644 761 people in each cohort (table 2). The majority of studies focused on all age groups (67%; n=8) and the mean age in the study population was 44.1 years, with a 45% female population, on average (online supplemental appendix 2). Almost all studies were rated as low risk of bias 75%; (75%; n=9) and the remaining were medium (25% n=3), with any concerns largely due to differences in comparison groups and length of follow-up. Most studies were population-based, covering the entirety of the population of people with intellectual and developmental disabilities. They descriptively assessed prevalence of the outcome for the disability and comparison groups, which made the JBI checklist questions on causal confounding less relevant and is why most did not have these factors.

**Table 1:** Study characteristics

Category	Characteristics of studies	N (%)
WHO region	African region	0
	Region of the Americas	2 (17)
	Eastern Mediterranean region	0
	European region	9 (75)
	South-East Asian region	0
	Western Pacific region	1 (8)
Study setting	Population or registry	12 (100)
Cohort sample size*	1–10 000	2 (13)
	10 001–100 000	1 (7)
	100 001–1 000 000	9 (60)
	1 000 000+	2 (13)
	Not reported	1 (7)
Number of people with disabilities in cohort*	1–1000	3 (20)
	1001–10 000	2 (13)
	10 000+	10 (67)
Disability type	Intellectual only	10 (83)
	Intellectual and developmental	2 (17)
	Vision	0
	Hearing	0
	Physical	0
	Psychosocial	0
Method of ascertaining disability status	Disability register	8 (64)
	ICD-10 codes in medical records	2 (17)
	Linked dataset (census or school records)	2 (17)
Definition of avoidable mortality	UK Office of National Statistics	8 (67)
	OECD	1 (8)
	Australian Institute of Health and Welfare	1 (8)
	Canadian Institute for Health Information	1 (8)
	Other code list	1 (8)
Female (mean, %)		45
Age (mean, years)		44.1
Follow-up time mean: 8.3 years; median: 5 years	0–4 years	5 (42)
	5–10 years	4 (33)
	10+ years	3 (20)
Quality rating (JBI Critical Appraisal)	Low risk of bias	9 (75)
	Medium risk of bias	3 (25)
	High risk of bias (score ≤7)	0

* Out of 15 included cohorts.

ICD-10, International Classification of Diseases, 10th Revision; JBI, Joanna Briggs Institute; OECD, Organisation for Economic Co-operation and Development.

**Table 2:** Study methods and demographics details

Author, country	Disability measure	Data source	Age group	Age (mean (SD)	% female	Number of people with disabilities	Number of people without disabilities	Total sample size
Cooper *et al*^42^, UK	GP learning disability register	Population cohort	All ages	44.1	45.3	961	–	961
Glover *et al*^43^, UK	GP learning disability register	UK GP data records (CPRD)	All ages	NR	50.6	14 787	2 775 213	2 790 000
Heslop *et al*^12^, UK	GP learning disability register	Death certificates	All ages (4+)	64 (IQR: 52–75)	42.1	247	480 467	480 714
		Comparative sub-set	All ages (4–74)	60 (IQR: 53–64)	50	58	58	116
Hirvikoski *et al*^16^, Sweden	Disability register	Cohort A—HURPID and NPR database	Young adults	24.5 (3.66)	42.2	13 541	135 410	148 951
		Cohort B mild—NPR	All ages	52.01 (16.88)	43.3	24 059	240 590	264 649
		Cohort C moderate profound—NPR	All ages	42.16 (21.68)	42.3	26 602	266 020	292 622
Hosking *et al*^38^, UK	GP learning disability register	UK GP data records (CPRD)	All ages	39.9 (16.2)	58	16 666	113 562	130 228
Hughes-McCormack *et al*^44^, UK	Census data (Scottish Learning Disabilities Observatory)	Population cohort identified from census	Children and young people (5–24)	NR	38.5	7247	156 439	163 686
Lin *et al*^18^, Canada	Administrative data and ICD-9 or ICD-10 codes	Population cohort	All ages	NR	42.7	64 699	2 683 532	2 748 231
Rydzewska *et al*^45^, UK	Census data	Population cohort identified from census	Adults (25+)	65 years (14.1)	45.2	14 477	506 207	520 684
Shooshtari *et al*^46^, Canada	Administrative data and ICD-9 or 10 codes	Population cohort	Adults (25+)	60 years (14)	45.3	6216	18 648	24 864
Smith *et al*^47^, UK	School records (annual pupil census)	Population cohort	Children and young people (5–24)	NR	45	18 278	777 912	796 190
Thygesen *et al*^15^, Denmark	Disability register	Population cohort	Adults (18–74)	NR	44.8	57 663	607 097	664 760
Trollor *et al*^37^, Australia	Disability service receipt (register)	Population cohort	Adults (20+)	37 (IQR 27–48)	44	19 362	NR	NR

GP, general practitioner; ICD-9, International Classification of Diseases, 9th Revision; ICD-10, International Classification of Diseases, 10th Revision; NR, not reported; OECD, Organisation for Economic Co-operation and Development.

### Avoidable mortality

In the 12 cohorts that reported relative measures, the meta-analysis showed that people with disabilities were 3.60 times more likely (figure 2: 95% CI 3.53 to 3.68) to die from avoidable causes compared with people without disabilities, although again there was high heterogeneity (I^2^=97%). All studies found that people with disabilities had a higher proportion of avoidable mortality compared with people without disabilities in the common effects model. The meta-analysis of 10 cohorts found that 45% (95% CI 45% to 46%) of mortality was avoidable for people with disabilities across 22 405 recorded deaths (table 3). By comparison, the proportion of avoidable mortality for people without disabilities was 19% (95% CI 19% to 19%) across eight studies and 488 414 deaths. Prevalence estimates ranged from 31% in Australia^37^ to 59% in Sweden^16^ for people with disabilities, while estimates for people with disabilities had a wider range, from 17% in Australia to 85% in Sweden in these same studies. Both meta-analyses had high heterogeneity (I^2^=98% and 100%, respectively), possibly due to more precise estimates from larger studies.

**Figure 2:**
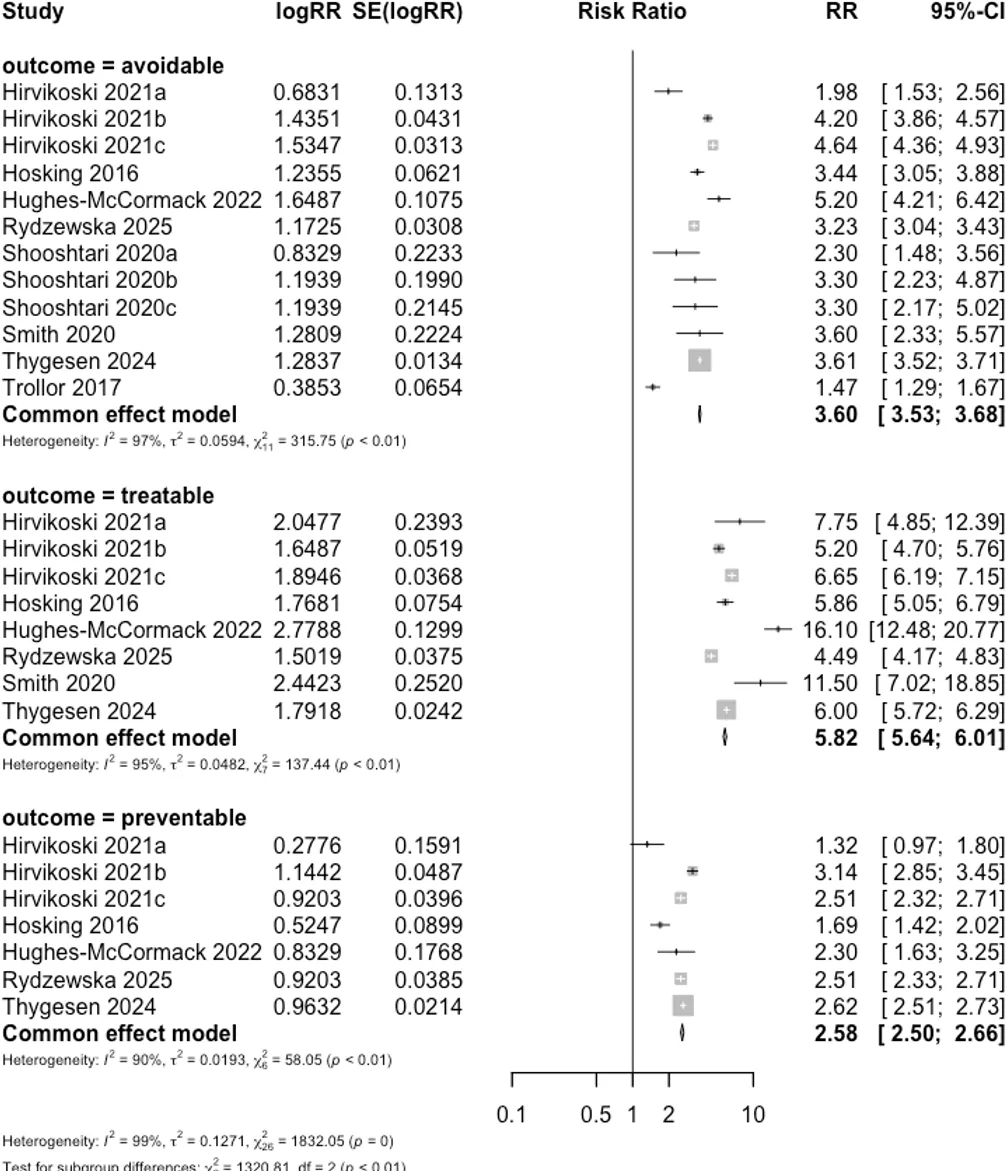
Relative risk (RR) of avoidable, treatable and preventable mortality for people with disabilities compared with people without disabilities.

**Table 3:** Summary results of meta-analysis

	Proportion estimate				Relative estimate
Outcome	People with disabilities sample size	Proportion among people without disabilities (95% CI)	People without disabilities sample size	Proportion among people with disabilities (95% CI)	Relative risk (95% CI)
Avoidable mortality	22 405	45% (45% to 46%)	488 414	19% (19% to 19%)	3.60 (3.53 to 3.68)
Treatable mortality	25 064	22% (21% to 22%)	127 988	11% (11% to 12%)	5.82 (5.64 to 6.01)
Preventable mortality	21 104	21% (20% to 21%)	85 329	18% (18% to 18%)	2.58 (2.50 to 2.66)

### Treatable mortality

Eight cohorts reported relative measures of treatable mortality. The pooled estimate showed that people with disabilities have 5.82 times (95% CI 5.64 to 6.01; I^2^=95%) higher treatable mortality compared with people without disabilities. The proportion of treatable mortality was reported in 11 cohorts for 25 064 deaths among people with disabilities. Overall, 22% (95% CI 21% to 22%; I2=98%) of these deaths were deemed treatable among people with disabilities, though this estimate ranged from 9% to 37% in two different UK studies.^12,38^ This proportion was substantially lower for people without disabilities (11%, 95% CI 11% to 12%; I^2^=100%) in 127 988 deaths, though this was measured in only five studies.

### Preventable mortality

Relative measures of preventable mortality were included in seven studies, yielding a pooled estimate of 2.58 times higher for people with disabilities (95% CI 2.50 to 2.66; I^2^=90%). Across 10 cohorts and 21 104 deaths, 21% (95% CI 20% to 21%) of deaths were preventable among people with disabilities. This proportion was similar among people without disabilities in seven cohorts (18%, 95% CI 18% to 18%) and 85 329 deaths. Heslop *et al* found only 4% of deaths were preventable,^12^ while Hirvikoski found 37% were among people with disabilities in Sweden.^16^

### Publication bias

Online supplemental appendix 6 reports the funnel plots for all outcomes. The funnel plots for all outcomes demonstrated asymmetry, which may indicate publication bias and a small number of studies. However, the underlying heterogeneity (>90%) may mean the asymmetry may reflect some differences between studies, rather than publication bias alone.

## Discussion

This is the first global systematic review on avoidable mortality and disability. We only identified studies on intellectual and developmental disabilities, and all available evidence comes from high-income countries. Our results provide clear evidence that people with intellectual and developmental disabilities experience a significantly higher proportion of avoidable mortality compared with people without disabilities. Avoidable mortality is a recognised measure that reflects difficulties in accessing timely, accessible and effective healthcare for people under 75 years old,^21^ rather than mortality associated with impairment. Data from this systematic review and meta-analysis show people with and without intellectual and developmental disabilities have high proportions of avoidable mortality (45% vs 19%), with substantial variation in estimates between studies, though the RRs from other studies indicate large inequities for people with disabilities. Avoidable mortality is three times greater among people with intellectual and developmental disabilities, and this association is over five times greater for preventable causes, suggesting the need for targeted primary care and public health interventions, including vaccination, screening and risk factor management. These are, of course, associations with substantial statistical heterogeneity (>90%) only from high-income countries and do not prove causation, but the consistent pattern suggests there needs to be greater attention to preventing avoidable mortality for people with disabilities within health systems. Moreover, these results also highlight that there are important evidence gaps on avoidable mortality for people with disabilities generally, as well as those living in low- and middle-income countries.

This study presents the first systematic review on disability and avoidable mortality. Mortality for people with disabilities has been shown to be higher than people with disabilities, which may be because of underlying impairment.^7^ However, these results provide new evidence suggesting that there is a large proportion of early mortality that is not associated with underlying impairment and these startling findings are in line with other studies and reports that show higher mortality for people with intellectual and developmental disabilities. Notably, the UK LeDeR annual reports show that people with intellectual disabilities have 40% avoidable mortality, which is twice that of the general population.^19,20^ The UK Confidential Inquiry in 2014 showed that many of these deaths are avoidable and occur because of health systems failures.^12^ For example, 67% of deaths among people with learning disabilities were due to delays in diagnosis, compared with 47% in the comparison group. Many of these deaths were also associated with challenges with accessibility of information and coordination, which were comparatively not important factors for death in the comparison groups.^12^ These data provide rich information into the mechanisms for early and avoidable mortality for people with disabilities, that may otherwise be missing in population-based studies. However, other data support the link between barriers to public health and primary prevention interventions and these outcomes. For example, breast and cervical cancer are considered preventable causes of mortality and a recent systematic review showed that women with disabilities have 22% and 35% lower odds of attending screening than women without disabilities, respectively.^39,40^ Similarly, cardiovascular diseases are more common among people with intellectual disabilities, but the evidence shows that they are less likely to receive treatment compared with people without disabilities.^41^ These gaps in preventive care for people with disabilities may contribute to the observed inequities in avoidable mortality, and provide further evidence that these associations are unlikely due to impairment alone. However, there are additional factors that need further research to contextualise these results fully. For example, health systems design or level of impairment may contribute to the underlying association, and so further research to understand these relationships may be important to ultimately reducing these inequalities.

### Strengths and limitations

This review has many notable strengths, including following the gold-standard approach to systematic review (eg, double screening, searching multiple databases), the consistency of disability measures and validated outcome definitions in included studies, and a meta-analysis of multiple studies. Moreover, most of the studies included were nationally representative and register-based, meaning the data represent nearly 10 million people in five countries. All studies included robust measures of disability (ie, being on a disability register), had long follow-up times (minimum 2 years) and comparable measures of avoidable mortality from a recognised entity (OECD or comparable Office of National Statistics definition).

However, there are several notable limitations. First, this is a global review but there were no studies conducted in low- and middle-income countries, where 80% of people with disabilities live, hampering our ability to draw conclusions at the global level. Second, most relative measures included in this study were unadjusted, and so may omit possible confounding between an individual’s impairment and higher mortality. This is a particular concern, since people with intellectual and developmental disabilities are younger, on average, and so these estimates may underestimate the true association. However, given that all studies focused on people with intellectual and developmental disabilities that have an early onset in life, most socioeconomic or comorbidities are likely mediators rather than confounders, though people. Third, substantial statistical heterogeneity was observed across the pooled analyses. The included studies varied markedly in cohort size for comparison groups. Most were large population-based datasets including over 100 000 participants, but some had small groups of under a few hundred people. These differences resulted in substantial variation in the precision of study estimates, such that even modest between-study differences contributed to high I² values. Consequently, the pooled estimates should be interpreted as average effects across diverse study populations rather than as a single common effect, and caution is warranted when generalising the findings to individual settings. Fourth, using a well-validated measure of avoidable mortality recognised by OECD and other national statistics offices strengthens the reliability of our results, but still may not account for the relationship between some underlying impairments and the outcomes (eg, poorer immune function could explain higher mortality from infectious diseases). There is no specific definition of avoidable mortality for people with disabilities, but further research could distill if there are specific causes of death that should or should not be deemed avoidable for this population. Fifth, no language restrictions were on our search, but we only found English literature, possibly missing additional studies from the non-English literature. Another notable limitation is the possibility of publication bias, as all funnel plots demonstrated asymmetry. However, this asymmetry should be interpreted cautiously, as it may also reflect small-study effects or the substantial between-study heterogeneity observed across the analyses, rather than publication bias alone. Moreover, our findings are limited to people with intellectual and developmental disabilities, as there are otherwise gaps in the literature on people with disabilities generally. This presents a methodological limitation, as people with intellectual and developmental disabilities make up just 1% of the population globally and a small proportion of people with disabilities. Finally, there are also important intersectional factors, such as sex, race/ethnicity and place of residence that further contribute to these possible inequities, but these studies did not report these disaggregated analyses.

## Conclusion

Avoidable mortality is an important measure of health equity for people with disabilities, given the barriers they experience in accessing health systems. It provides an important indicator to distinguish how the health system contributes to the 14-year life expectancy gap observed for people with disabilities. More studies are required to strengthen the evidence on avoidable mortality for people with disabilities globally. Yet, there is ample evidence of avoidable mortality for people with intellectual disabilities from a variety of settings showing disproportionately higher rates. Possible interventions could be derived from further analysis of the causes of these early deaths and systems failures that led to these deaths. The magnitude of these inequities should make improving health services for people with intellectual disabilities a key government priority globally.

## Supplementary material

10.1136/bmjph-2025-004826 online supplemental appendix 1

10.1136/bmjph-2025-004826 online supplemental appendix 2

10.1136/bmjph-2025-004826 online supplemental appendix 3

10.1136/bmjph-2025-004826 online supplemental appendix 4

10.1136/bmjph-2025-004826 online supplemental appendix 5

10.1136/bmjph-2025-004826 online supplemental appendix 6

## Data Availability

Data are available upon reasonable request.
