# Supplementary material for: Avoidable mortality for people with disabilities: a systematic review and meta-analysis
Source: BMJ Public Health. 2026 Oct 5;4(4):e004826. doi: 10.1136/bmjph-2025-004826 (PMC13646978; doi:10.1136/bmjph-2025-004826)
Supplement: Supplementary file 1 [file bmjph-4-4-s001.docx]

Appendix 1: Search Strategy for OVID Platform

| Search Line | Search terms |  |
| --- | --- | --- |
| 88 | remove duplicates from 87 |  |
| 87 | limit 86 to yr="2000 -Current" |  |
| 86 | 79 and 85 |  |
| 85 | 80 or 81 or 82 or 83 or 84 |  |
| 84 | (excess adj1 (mortal* or fatal* or death*)).ti,ab. |  |
| 83 | (amenable adj1 (mortal* or fatal* or death*)).ti,ab. |  |
| 82 | (avoid* adj1 (mortal* or fatal* or death*)).ti,ab. |  |
| 81 | (treat* adj1 (mortal* or fatal* or death*)).ti,ab. |  |
| 80 | (prevent* adj1 (mortal* or fatal* or death*)).ti,ab. |  |
| 79 | 1 or 2 or 3 or 4 or 5 or 6 or 7 or 8 or 9 or 10 or 11 or 12 or 13 or 14 or 15 or 16 or 17 or 18 or 19 or 20 or 21 or 22 or 23 or 24 or 25 or 26 or 27 or 28 or 29 or 30 or 31 or 32 or 33 or 34 or 35 or 36 or 37 or 38 or 39 or 40 or 41 or 42 or 43 or 44 or 45 or 46 or 47 or 48 or 49 or 50 or 51 or 52 or 53 or 54 or 55 or 56 or 57 or 58 or 59 or 60 or 61 or 62 or 63 or 64 or 65 or 66 or 67 or 68 or 69 or 70 or 71 or 72 or 73 or 74 or 75 or 76 or 77 or 78 |  |
| 78 | Dysarthria.ti,ab. |  |
| 77 | (language adj1 (disab* or handicap* or impair* or abnormal* or defect*)).ti,ab. |  |
| 76 | (speech adj1 (disab* or handicap* or impair* or abnormal* or defect*)).ti,ab. |  |
| 75 | (communicat* adj1 (disab* or handicap* or impair* or abnormal* or defect*)).ti,ab. |  |
| 74 | Communication Disorders/ |  |
| 73 | neurodivers*.ti,ab. |  |
| 72 | ADHD.ti,ab. |  |
| 71 | attention deficit hyperactivity disorder.ti,ab. |  |
| 70 | pervasive development* disorder*.ti,ab. |  |
| 69 | heller* syndrome.ti,ab. |  |
| 68 | rett syndrome.ti,ab. |  |
| 67 | asperger*.ti,ab. |  |
| 66 | down* syndrome.ti,ab. |  |
| 65 | fragile x syndrome.ti,ab. |  |
| 64 | f?etal alcohol syndrome.ti,ab. |  |
| 63 | autis*.ti,ab. |  |
| 62 | child development disorders, pervasive/ |  |
| 61 | (development* adj1 (disab* or handicap* or impair* or abnormal* or defect*)).ti,ab. |  |
| 60 | Developmental Disabilities/ |  |
| 59 | (learning adj1 (disab* or handicap* or impair* or abnormal* or defect*)).ti,ab. |  |
| 58 | learning disabilities/ |  |
| 57 | (intellectual* adj1 (disab* or handicap* or impair* or abnormal* or defect*)).ti,ab. |  |
| 56 | Intellectual Disability/ |  |
| 55 | partial* sight*.ti,ab. |  |
| 54 | (blind not single blind* not double blind* not triple blind* not blinding not blinded not blindly).ti,ab. |  |
| 53 | (visual* adj1 (disab* or handicap* or impair* or abnormal* or defect*)).ti,ab. |  |
| 52 | (vision adj1 (disab* or handicap* or impair* or abnormal* or defect*)).ti,ab. |  |
| 51 | Vision Disorders/ |  |
| 50 | persons with hearing impairments/ |  |
| 49 | deaf*.ti,ab. |  |
| 48 | (hard adj2 hearing).ti,ab. |  |
| 47 | (hearing adj1 (loss or disab* or handicap* or impair* or abnormal* or defect*)).ti,ab. |  |
| 46 | hearing disorders/ |  |
| 45 | hearing loss/ |  |
| 44 | Microcephaly.ti,ab. |  |
| 43 | (restricted adj1 (growth or stature)).ti,ab. |  |
| 42 | dwarf*.ti,ab. |  |
| 41 | Achondroplasia.ti,ab. |  |
| 40 | Dwarfism/ |  |
| 39 | albin*.ti,ab. |  |
| 38 | Albinism/ |  |
| 37 | wheelchair user*.ti,ab. |  |
| 36 | (spin* adj1 injur*).ti,ab. |  |
| 35 | Hemiplegi*.ti,ab. |  |
| 34 | quadriplegi*.ti,ab. |  |
| 33 | Paraplegi*.ti,ab. |  |
| 32 | paraly*.ti,ab. |  |
| 31 | Paralysis/ |  |
| 30 | polio*.ti,ab. |  |
| 29 | Poliomyelitis/ |  |
| 28 | ataxia.ti,ab. |  |
| 27 | Ataxia/ |  |
| 26 | clubfoot.ti,ab. |  |
| 25 | Clubfoot/ |  |
| 24 | amput*.ti,ab. |  |
| 23 | MND.ti,ab. |  |
| 22 | motor neurone disease.ti,ab. |  |
| 21 | Motor Neuron Disease/ |  |
| 20 | ALS.ti,ab. |  |
| 19 | Amyotrophic lateral sclerosis.ti,ab. |  |
| 18 | (musculoskeletal adj1 (abnormal* or disorder* or condition* or disab* or impair*)).ti,ab. |  |
| 17 | Musculoskeletal Abnormalities/ |  |
| 16 | Osteogenesis Imperfecta.ti,ab. |  |
| 15 | Osteogenesis Imperfecta/ |  |
| 14 | (muscular adj1 (abnormal* or disorder* or condition* or disab* or impair* or dystroph*)).ti,ab. |  |
| 13 | Muscular Disorders, Atrophic/ |  |
| 12 | spina bifida.ti,ab. |  |
| 11 | spinal dysraphism*.ti,ab. |  |
| 10 | Spinal Dysraphism/ |  |
| 9 | cerebral palsy.ti,ab. |  |
| 8 | Cerebral Palsy/ |  |
| 7 | ((congenital or genetic) adj1 (disab* or handicap* or impair* or abnormal* or defect*)).ti,ab. |  |
| 6 | (physical* adj1 (disab* or handicap* or impair* or abnormal* or defect*)).ti,ab. |  |
| 5 | (special adj1 need*).ti,ab. |  |
| 4 | ((people or person* or adult* or men or women or child* or youth or adolescent* or girl* or boy*) adj1 impair*).ti,ab. |  |
| 3 | ((people or person* or adult* or men or women or child* or youth or adolescent* or girl* or boy*) adj1 handicap*).ti,ab. |  |
| 2 | ((people or person* or adult* or men or women or child* or youth or adolescent* or girl* or boy*) adj1 disab*).ti,ab. |  |
| 1 | exp Disabled Persons/ |  |
