# Supplementary material for: Avoidable mortality for people with disabilities: a systematic review and meta-analysis
Source: BMJ Public Health. 2026 Oct 5;4(4):e004826. doi: 10.1136/bmjph-2025-004826 (PMC13646978; doi:10.1136/bmjph-2025-004826)
Supplement: Supplementary file 2 [file bmjph-4-4-s003.docx]

| **Appendix 3: JBI Quality Appraisal Tool** | | | | | | | | | | | | | |
| --- | --- | --- | --- | --- | --- | --- | --- | --- | --- | --- | --- | --- | --- |
| **Study Author (Year)** | **1. Were the two groups similar and recruited from the same population?** | **2. Were the exposures measured similarly to assign people to both exposed and unexposed groups?** | **3. Was the exposure measured in a valid and reliable way?** | **4. Were confounding factors identified?** | **5. Were strategies to deal with confounding factors stated?** | **6. Were the groups or participants free of the outcome at the start of the study (or at the moment of exposure)?** | **7. Were the outcomes measured in a valid and reliable way?** | **8. Was the follow up time reported and sufficient to be long enough for outcomes to occur?** | **9. Was follow up complete, and if not, were the reasons to loss to follow up described and explored?** | **10. Were strategies to address incomplete follow up utilized?** | **11. Was appropriate statistical analysis used?** | **Quality Rating** |  |
| Cooper 2020 | Yes | Partially | Yes | No | No | Yes | Yes | Yes | Yes | No | Yes | Medium |  |
| Glover 2017 | Yes | Yes | Yes | No | No | Yes | Yes | Yes | Yes | No | Yes | High |  |
| Heslop 2014 | Yes | Yes | Yes | Yes | Yes | Yes | Yes | Yes | Yes | No | Yes | High |  |
| Hirvikoski 2021 | Yes | Yes | Yes | Yes | Yes | Yes | Yes | Yes | Yes | No | Yes | High |  |
| Hosking 2016 | Yes | Yes | Yes | No | No | Yes | Yes | Yes | Yes | No | Yes | High |  |
| Hughes-McCormack 2022 | Yes | Yes | Yes | No | No | Yes | Yes | Yes | Yes | No | Yes | High |  |
| Lin 2023 | Yes | Yes | Yes | No | No | Yes | Yes | Partially | Yes | No | Yes | Medium |  |
| Rydzewska 2025 | Yes | Yes | Yes | No | No | Yes | Yes | Yes | Yes | No | Yes | High |  |
| Shooshtari 2020 | Yes | Yes | Yes | No | No | Yes | Yes | Partially | Yes | No | Yes | Medium |  |
| Smith 2020 | Yes | Yes | Yes | No | No | Yes | Yes | Yes | Yes | No | Yes | High |  |
| Thygesen 2024 | Yes | Yes | Yes | Yes | Yes | Yes | Yes | Yes | Yes | No | Yes | High |  |
| Trollor 2017 | Yes | Partially | Yes | Yes | Yes | Yes | Yes | Partially | Yes | No | Yes | High |  |
