# Supplementary material for: Avoidable mortality for people with disabilities: a systematic review and meta-analysis
Source: BMJ Public Health. 2026 Oct 5;4(4):e004826. doi: 10.1136/bmjph-2025-004826 (PMC13646978; doi:10.1136/bmjph-2025-004826)
Supplement: Supplementary file 4 [file bmjph-4-4-s005.docx]

**Appendix 5: Reasons for exclusion summary table**

| Title | Study | Reason for exclusion |
| --- | --- | --- |
| Investigating deaths of people with autistic spectrum disorder in health and social care in England and Wales. | Norris 2025 | No full text |
| Contributing causes of mortality and potentially avoidable deaths of people with intellectual or learning disability: A data-linkage study. | Iffland 2024 | Wrong outcomes |
| How people with intellectual disability are dying and implications for quality care. | Bernal 2022 | Wrong study design |
| Health checks. | Lennox 2014 | Wrong study design |
| Survival of children and young people with cerebral palsy. | Srivastava 2011 | Wrong study design |
| Preventable deaths from SARS-CoV-2 in England and Wales: A systematic case series of coroners' reports during the COVID-19 pandemic | Swift 2021 | Wrong study design |
| Population-based cancer incidence and mortality rates and ratios among adults with intellectual disabilities in Scotland | Ward 2024 | Wrong outcomes |
| Disease and mortality trajectories of cognitively able autistic individuals in mid- and later adulthood | Pu 2025 | Wrong outcomes |
| Excess mortality among patients with severe mental disorders and effects of community-based mental healthcare: A community-based prospective study in Sichuan, China | Li 2021 | Wrong outcomes |
| Additive interaction of disability with chronic conditions on mortality risk in middle-aged and older adults in Spain | Pastor-Barriuso 2024 | Wrong outcomes |
| Excess mortality and suicide risk in treatment-seeking individuals with schizotypal disorder: A population-based study using Swedish national registers | Berge 2024 | Wrong outcomes |
| Excess Deaths in Assisted Living and Nursing Homes during the COVID-19 Pandemic in Alberta, Canada | Hogan 2024 | Wrong outcomes |
| Overall and cause-specific mortality in anorexia nervosa; impact of psychiatric comorbidity and sex in a 40-year follow-up study | Soeby 2024 | Wrong patient population |
| Excess Mortality in Individuals with Autism Spectrum Disorder: A Population-Based Cohort Study | Huang 2024 | Wrong outcomes |
| Medical care use and mortality rate after the onset of disability: A 6-year follow-up study based on national data in Taiwan | Chen 2024 | Wrong outcomes |
| Long term mortality trends in people with severe mental illnesses and how COVID-19, ethnicity and other chronic mental health comorbidities contributed: a retrospective cohort study | Das-Munshi 2024 | Wrong outcomes |
| Life years lost associated with mental illness: A cohort study of beneficiaries of a South African medical insurance scheme | Ruffieux 2023 | Wrong outcomes |
| All-cause and cause-specific mortality among people with and without intellectual disabilities during the COVID-19 pandemic in the Netherlands: a population-based cohort study | Cuypers 2023 | Wrong outcomes |
| Exploring the Factors Associated with Avoidable Deaths of People with an Intellectual Disability in England in 2021 | Tuffrey-Wijne 2023 | Wrong study design |
| COVID-19 infection and outcomes in a population-based cohort of 17 203 adults with intellectual disabilities compared with the general population | Henderson 2022 | Wrong outcomes |
| Mortality in Persons with Autism Spectrum Disorder or Attention-Deficit/Hyperactivity Disorder: A Systematic Review and Meta-analysis | Catala-Lopez 2022 | Wrong study design |
| Predictors of hospitalisation and death due to SARS-CoV-2 infection in Finland: A population-based register study with implications to vaccinations | Salo 2022 | Wrong outcomes |
| Inequitable distribution of excess mortality during the COVID-19 pandemic in Korea, 2020 | Kim 2022 | Wrong outcomes |
| Factors associated with excess all-cause mortality in the first wave of the COVID-19 pandemic in the UK: A time series analysis using the Clinical Practice Research Datalink | Strongman 2022 | Wrong outcomes |
| Cohort profile: Scotland's record-linkage e-cohorts of people with intellectual disabilities, and autistic people (SCIDA) | Cooper 2022 | Wrong study design |
| A national study of epilepsy-related deaths in Scotland: Trends, mechanisms, and avoidable deaths | Mbizvo 2021 | Wrong outcomes |
| Understanding inequalities in COVID-19 outcomes following hospital admission for people with intellectual disability compared to the general population: A matched cohort study in the UK | Baksh 2021 | Wrong outcomes |
| Excess deaths in treatment-resistant depression | Brenner 2021 | Wrong outcomes |
| Risk factors for COVID-19 diagnosis, hospitalization, and subsequent all-cause mortality in Sweden: a nationwide study | Bergman 2021 | Wrong outcomes |
| Risk factors for excess all-cause mortality during the first wave of the COVID-19 pandemic in England: A retrospective cohort study of primary care data | Carey 2021 | Wrong outcomes |
| Excess mortality in the first COVID pandemic peak: Cross-sectional analyses of the impact of age, sex, ethnicity, household size, and long-term conditions in people of known SARS-CoV-2 status in England | Joy 2020 | Wrong outcomes |
| Mortality of people with intellectual disabilities during the 2017/2018 influenza epidemic in the Netherlands: potential implications for the COVID-19 pandemic | Cuypers 2020 | Wrong outcomes |
| Mortality in people living with HIV and mental health disorders in South Africa | Haas 2020 | Wrong outcomes |
| Mortality and cause of death of Australians on the autism spectrum | Hwang 2019 | Wrong outcomes |
| Health and wellbeing of people with intellectual disability in New South Wales, Australia: A data linkage cohort | Reppermund 2019 | Wrong study design |
| Patterns of mortality among adults with intellectual and developmental disabilities in Ontario | Stankiewicz 2019 | Wrong outcomes |
| Closing the medical mortality gap in patients with major psychiatric conditions | deSilva 2018 | Wrong study design |
| Excess mortality from multiple sclerosis among men in the armed forces in England and Wales during 1979-2010 | Harris 2016 | Wrong outcomes |
| Mortality in children, adolescents, and adults with attention deficit hyperactivity disorder: A nationwide cohort study | Dalsgaard 2015 | Wrong outcomes |
| A 13-year follow-up of Finnish patients with Salla disease | Paavola 2015 | Wrong outcomes |
| Mortality among a Cohort of Persons with an Intellectual Disability in New South Wales, Australia | Florio 2015 | Wrong outcomes |
| Understanding Information About Mortality Among People with Intellectual and Developmental Disabilities in Canada | Ouellette-Kuntz 2015 | Wrong study design |
| Co-morbidity and mortality in alzheimer's disease | Schoepf 2015 | Wrong study design |
| Causes of death following spinal cord injury during inpatient rehabilitation and the first five years after discharge. A Dutch cohort study | Osterthun 2014 | Wrong outcomes |
| Excess mortality after disability retirement due to mental disorders: variations by socio-demographic factors and causes of death | Leinonen 2014 | Wrong outcomes |
| Comparative mortality of persons with intellectual disability in California: an update (2000-2010) | Shavelle 2014 | No full text |
| What do ALS patients die of?-an autopsy study of 70 ALS patients | Burkhardt 2014 | Wrong study design |
| Excess mortality and causes of death in autism spectrum disorders: A follow up of the 1980s Utah/UCLA autism epidemiologic study | Bilder 2013 | Wrong outcomes |
| Alcohol-related deaths and social factors in depression mortality: A register-based follow-up of depressed in-patients and antidepressant users in Finland | Moustgaard 2013 | Wrong outcomes |
| Safety of patients with intellectual disabilities in UK hospitals | Tuffrey-Wijne 2012 | Wrong outcomes |
| Excess mortality in osteoarthritis | Cooper 2011 | Wrong patient population |
| "Permanent discharge": deaths of people under 50 years of age in residential aged care in Victoria | Dearn 2011 | Wrong study design |
| Cause of death for children and adolescents with a spinal cord injury | Zebracki 2011 | Wrong outcomes |
| Cause-specific mortality and death certificate reporting in adults with moderate to profound intellectual disability | Tyrer 2009 | Wrong outcomes |
| Inhospital mortality of children with down syndrome | Wisotsky 2010 | Wrong study design |
| Mortality and causes of death in autism spectrum disorders: An update | Mouridsen 2008 | Wrong outcomes |
| Vaccine preventable deaths and the Global Immunization Vision and Strategy | Anonymous 2006 | Wrong patient population |
| Were less disabled patients the most affected by 2003 heat wave in nursing homes in Paris, France? | Holstein 2005 | Wrong outcomes |
| Mortality and causes of death in persons with Down syndrome in California | Day 2005 | Wrong outcomes |
| Long-term mortality after poliomyelitis | Nielsen 2003 | Wrong outcomes |
| Mortality and cancer incidence among individuals with Down syndrome | Hill 2003 | Wrong outcomes |
| Remote symptomatic epilepsy: Does seizure severity increase mortality? | Strauss 2003 | Wrong outcomes |
| Causes of Death in Autism | Shavelle 2001 | Wrong outcomes |
| Excess mortality associated with blindness in the onchocerciasis focus of the Mbam Valley, Cameroon | Pion 2002 | Wrong outcomes |
| Mortality in various types of osteogenesis imperfecta | Singer 2001 | Wrong outcomes |
| Patterns of mortality among people with autism spectrum disorder in South Korea. | Kim 2025 | Wrong outcomes |
| Ten-year trend analysis of mortality due to external causes of injury in people with disabilities, South Korea, 2008-2017. | Kim 2021 | Wrong outcomes |
| Identifying the factors affecting the implementation of strategies to promote a safer environment for patients with learning disabilities in NHS hospitals: a mixed-methods study | Tuffrey-Wijne 2013 | Wrong study design |
| All-cause and cause-specific mortality in people with mental disorders and intellectual disabilities, before and during the COVID-19 pandemic: cohort study. | Das-Munshi 2021 | Wrong outcomes |
| Survival and causes of death in adults with spina bifida in Sweden: a population-based case-control study. | Rocchi 2023 | Wrong outcomes |
| The mortality experience of disabled persons in the United States during the COVID-19 pandemic. | Weaver 2024 | Wrong outcomes |
| [Considerations on Excess Mortality in Germany in the year 2020 and 2021]. | Baum 2022 | Wrong outcomes |
| Mortality in mental health patients of the Emilia-Romagna region of Italy: A registry-based study. | Berardi 2021 | Wrong outcomes |
| Mortality and Medical Comorbidity in the Severely Mentally Ill. | Schneider 2019 | Wrong outcomes |
| Mortality Rates and Excess Death Rates for the Seriously Mentally Ill. | Reynolds 2018 | Wrong outcomes |
| Understanding excess mortality in persons with mental illness: 17-year follow up of a nationally representative US survey. | Druss 2011 | Wrong outcomes |
| Adverse events and potentially preventable deaths in Dutch hospitals: results of a retrospective patient record review study. | Zegers 2009 | Wrong patient population |
| Mortality in long-stay patients from psychiatric hospitals in Italy--results from the Qualyop Project. | D'Avanzo 2003 | Wrong outcomes |
| Excess mortality among long-stay psychiatric patients in Northern Finland. | Rasanen 2003 | Wrong outcomes |
| "A completely preventable death". | Sibbald 2002 | Wrong study design |
| Causes of excess mortality in cerebral palsy. | Maudsley 2000 | Wrong study design |
| Expecting the unexpected: measures, outcomes and dying trajectories for expected and unexpected death in adults with intellectual disabilities in social care settings in the UK | Bernal 2021 | Wrong outcomes |
| Better health and care for all: health and care services for people with learning disabilities | NIHR 2020 | Wrong study design |
| Premature deaths of people with learning disabilities: progress update | Department of Health 2014 | Wrong study design |
| Mortality of people with intellectual disability in northern Sydney | Baker 2002 | Wrong study design |
| Mortality Among Youth and Young Adults With Autism Spectrum Disorder, Intellectual Disability, or Cerebral Palsy | Shaw 2026 | Wrong outcomes |
| All-cause and cause-specific mortality in people with mental disorders: a population-based study on risk evaluation, effect modifiers and excess life-years lost in Hong Kong | Chan 2026 | Wrong outcomes |
