# Supplementary material for: Avoidable mortality for people with disabilities: a systematic review and meta-analysis
Source: BMJ Public Health. 2026 Oct 5;4(4):e004826. doi: 10.1136/bmjph-2025-004826 (PMC13646978; doi:10.1136/bmjph-2025-004826)
Supplement: Supplementary file 5 [file bmjph-4-4-s006.docx]

**Appendix 6: Supplementary Figures**

Supplementary Figure 1: Proportion of avoidable mortality amongst people with and without disabilities

Supplementary Figure 2: Proportion of treatable mortality amongst people with and without disabilities

Supplementary Figure 3: Proportion of preventable mortality amongst people with and without disabilities

Supplementary Figure 4: Proportion of avoidable mortality meta-analysis funnel plot

Supplementary Figure 5: Proportion of treatable mortality meta-analysis funnel plot

Supplementary Figure 6: Proportion of preventable mortality meta-analysis funnel plot

Supplementary Figure 7: Relative inequalities in avoidable, preventable, and treatable mortality meta-analysis funnel plot
